# The Modulation of Mucosal Antiviral Immunity by Immunobiotics: Could They Offer Any Benefit in the SARS-CoV-2 Pandemic?

**DOI:** 10.3389/fphys.2020.00699

**Published:** 2020-06-16

**Authors:** Julio Villena, Haruki Kitazawa

**Affiliations:** ^1^Laboratory of Immunobiotechnology, Reference Centre for Lactobacilli (CERELA-CONICET), San Miguel de Tucumán, Argentina; ^2^Food and Feed Immunology Group, Laboratory of Animal Products Chemistry, Graduate School of Agricultural Science, Tohoku University, Sendai, Japan; ^3^Livestock Immunology Unit, International Education and Research Center for Food Agricultural Immunology (CFAI), Graduate School of Agricultural Science, Tohoku University, Sendai, Japan

**Keywords:** immunobiotics, respiratory viral infections, inflammation, coronavirus, beneficial microbes

## Abstract

Viral respiratory infections are of major importance because of their capacity to cause of a high degree of morbidity and mortality in high-risk populations, and to rapidly spread between countries. Perhaps the best example of this global threat is the infectious disease caused by the new SARS-CoV-2 virus, which has infected more than 4 million people worldwide, causing the death of 287,000 persons according to the WHO's situation report on May 13, 2020. The availability of therapeutic tools that would be used massively to prevent or mitigate the detrimental effects of emerging respiratory viruses on human health is therefore mandatory. In this regard, research from the last decade has reported the impact of the intestinal microbiota on the respiratory immunity. It was conclusively demonstrated how the variations in the intestinal microbiota affect the responses of respiratory epithelial cells and antigen presenting cells against respiratory virus attack. Moreover, the selection of specific microbial strains (immunobiotics) with the ability to modulate immunity in distal mucosal sites made possible the generation of nutritional interventions to strengthen respiratory antiviral defenses. In this article, the most important characteristics of the limited information available regarding the immune response against SARS-CoV-2 virus are revised briefly. In addition, this review summarizes the knowledge on the cellular and molecular mechanisms involved in the improvement of respiratory antiviral defenses by beneficial immunobiotic microorganisms such as *Lactobacillus rhamnosus* CRL1505. The ability of beneficial microorganisms to enhance type I interferons and antiviral factors in the respiratory tract, stimulate Th1 response and antibodies production, and regulate inflammation and coagulation activation during the course of viral infections reducing tissue damage and preserving lung functionally, clearly indicate the potential of immunobiotics to favorably influence the immune response against SARS-CoV-2 virus.

## Introduction

A severe respiratory disease emerged in China in December 2019 and spread globally infecting more than 4 million people worldwide and causing the death of 287,000 persons according to the WHO's situation report on May 13, 2020. This new infectious disease was designated coronavirus disease 2019 (COVID-19) and a virus from the coronavirus family was found to be the causal agent of this pathology (WHO, coronavirus pandemic). The virus was initially named 2019 new coronavirus (2019-nCoV) but later renamed by the International Committee on Taxonomy of Viruses as Severe Acute Respiratory Syndrome Coronavirus 2 (SARS-CoV-2) (Coronaviridae Study Group of the International Committee on Taxonomy of Viruses, 2020). Since the WHO's first situation report of SARS-CoV-2 infection on January 21, 2020, public health systems and scientists have rapidly learned about the transmission and mortality rates, the clinical manifestations as well as the immune responses although a more complete and precise understanding of the aspects of the COVID-19 pathology could take several more months.

**Figure 1:**
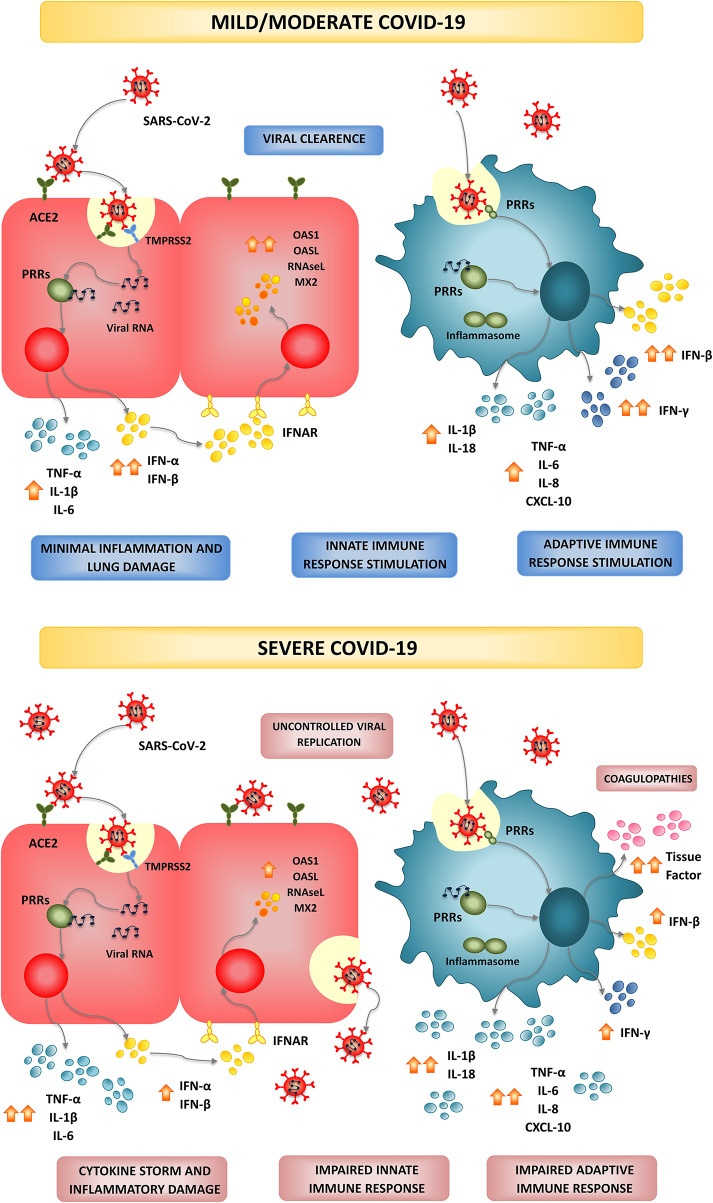
Infection and modulation of the immune system by SARS-CoV-2. In most individuals, SARS-CoV-2 infection triggers and efficient and timely production of type I IFNs and inflammatory cytokines by epithelial cells and immune cells creating an antiviral state and inducing the recruitment of additional immune cells that collaborate to clear the infection in the lung, with minimal inflammation and damage. This type of immune response is associated to mild or moderate forms of COVID-19 and patients finally recover. In high-risk populations such as the elderly and persons with comorbidities, a dysfunctional immune response is triggered by SARS-CoV-2 infection. A severe type of COVID-19 characterized by a cytokine storm that mediates widespread lung inflammation, coagulopathies, organ failure, and death occurs in some patients.

SARS-CoV-2 is an enveloped virus with a positive-sense, single-stranded RNA genome of ~30 kb, which shares some sequence homology with Severe Acute Respiratory Syndrome Coronavirus (SARS-CoV) (79% homology) and Middle East Respiratory Syndrome Coronavirus (MERS-CoV) (50% homology). The spike protein (also known as S protein) of coronaviruses are involved in the viral entry into target cells. The binding of the S protein to a specific cellular receptor allow the viral attachment to the surface of target cells while the S protein priming by cellular proteases facilitates the fusion of viral and cellular membranes (Figure 1). Bioinformatic analysis of the viral genome as well as *in vitro* and *in vivo* experiments demonstrated that the human angiotensin-converting enzyme 2 (ACE2) acts as the cell receptor for SARS-CoV-2 (Bao et al., 2020; Hoffmann et al., 2020a; Kim et al., 2020; Wang Q. et al., 2020), while the serine protease TMPRSS2 is involved in the S protein priming (Hoffmann et al., 2020b). In normal human lungs, ACE2 is expressed mainly in alveolar epithelial type II cells (or type II pneumocytes), which are the cells responsible of producing the surfactant that reduces surface tension and prevents the collapse of alveoli (Dobbs, 1989). Therefore, the destruction of type II pneumocytes by SARS-CoV-2 infection affects this critical function of respiratory cells impairing the gas exchange function of the lung (Zhu et al., 2020). There is also evidence of replication of SARS-CoV-2 in the upper respiratory tract since the inoculation of this virus to surface layers of human airway epithelial cells *in vitro* causes cytopathic effects and cessation of the cilium beating of the cells (Zhu et al., 2020). On the other hand, it was reported that SARS-Co-V directly infects macrophages and T cells, a key feature in SARS-CoV-mediated pathogenesis (Perlman and Dandekar, 2005; Shieh et al., 2005). While a recent report suggested that SARS-CoV-2 can directly infect T cells through S protein-mediated membrane fusion (Wang X. et al., 2020), whether the virus is capable of infecting other immune cells is still unknown. Studies reported that the ACE2 is also expressed in several extrapulmonary tissues including heart, kidneys, blood vessels, and intestine (Crackower et al., 2002; Ding et al., 2004; Danilczyk and Penninger, 2006). This fact could explain at least partially the multiorgan dysfunction observed in patients with severe COVID-19 (Guan et al., 2020; Huang et al., 2020). Of note, another receptor, CD147, has been implicated in mediating host cell invasion by SARS-CoV-2 (Wang K. et al., 2020). However, the role of CD147 and SARS-CoV-2 interaction in the pathology of COVID-19 needs further research.

SARS-CoV-2 binds to its receptor and enters the cells through endocytosis (Figure 1). Then, viral RNA is released into the cytosol and the virus exploits the cell machinery to replicate. Finally, SARS-CoV-2 is excreted from the cell by exocytosis. The rapid viral replication cause massive epithelial cell apoptosis, vascular leakage, and induce the release of pro-inflammatory cytokines and the recruitment of inflammatory cells (Jamilloux et al., 2020). The control of the viral replication and the efficient regulation of the inflammatory response determine the outcome of this infectious disease (Figure 1; Jamilloux et al., 2020; Merad and Martin, 2020; Tay et al., 2020). The majority of the persons infected with SARS-CoV-2 (80%) exhibit mild to moderate symptoms, while approximately 15% progress to severe pneumonia and 5% develop acute respiratory distress syndrome (ARDS), septic shock, and/or multiple organ failure (Chan et al., 2020; Ding et al., 2020; Huang et al., 2020; Prompetchara et al., 2020; Wu et al., 2020).

A high-quality clinical study performed by a group of clinicians and scientists from the University of Hong Kong in the city of Shenzhen provided several important clinical features of COVID-19 (Chan et al., 2020), which were later confirmed by several clinical trials around the world (Bai et al., 2020; Ding et al., 2020; Huang et al., 2020; Prompetchara et al., 2020; Wu et al., 2020). This pioneering study and the subsequent clinical investigations found a series of characteristics of COVID-19 that can be summarized as follows: (a) a high transmissibility of SARS-CoV-2 within the family context providing concrete evidence for human-to-human transmission, (b) variable clinical manifestations ranging from mild respiratory symptoms to severe ARDS and multiple organ failure, (c) more severe clinical abnormalities seen in older patients and/or patients with other co-morbidities (diabetes, hypertension, cardiovascular diseases), (d) the presence of SARS-CoV-2 RNA in samples of asymptomatic persons highlighting the possibility for transmission of the virus from asymptomatic carriers and, (e) the presence of gastrointestinal symptoms in some persons suggesting the possibility for gastrointestinal involvement in SARS-CoV-2 infection and fecal-oral transmission.

The most common symptoms of COVID-19 include respiratory symptoms such as cough, sore throat, shortness of breath, and pneumonia, as well as fever, fatigue, and in very few cases diarrhea and vomiting (Chan et al., 2020; Prompetchara et al., 2020). Secondary infections have been also reported to be involved in severe and fatal cases of SARS-CoV-2 infection (Huang et al., 2020). Around 10% of the fatal cases of COVID-19 have identified co-infections of SARS-CoV-2 with Influenza Virus (IFV) (Ding et al., 2020; Huang et al., 2020; Wu et al., 2020) and respiratory bacterial pathogens (Wang J. et al., 2020). Importantly, the role of the immune responses in the SARS-CoV-2 infection-induced lung and systemic damage has been highlighted by recent studies (Cao, 2020; Jamilloux et al., 2020; Tay et al., 2020). The information regarding the immune responses to SARS-CoV-2 was the result of limited investigations and mainly from extrapolations using insights learned from the outbreaks of SARS-CoV and MERS-CoV. Both innate and adaptive immune responses are stimulated by SARS-CoV-2, however; uncontrolled inflammatory responses and impaired adaptive immunity are characteristics of COVID-19 (Cao, 2020; Merad and Martin, 2020; Tay et al., 2020). The infiltration of inflammatory cells in the lung, lymphopenia, exhausted lymphocytes, and a cytokine storm actively participate in tissue damage, both locally and systemically.

The availability of therapeutic tools that could be used massively to prevent or mitigate the detrimental effects of emerging respiratory viruses such as SARS-CoV-2 on human health is therefore mandatory. In this regard, research from the last decade has reported the impact of the intestinal microbiota on the respiratory immunity. It has been conclusively demonstrated how variations in the intestinal microbiota can affect the responses of respiratory epithelial cells and antigen presenting cells against respiratory virus attack, influencing innate immune responses, and the subsequent adaptive immunity. Moreover, the selection of specific microbial strains with the ability to modulate immunity in distal mucosal sites made possible the generation of nutritional interventions to strengthen respiratory antiviral defenses (Villena et al., 2019). In this article, the most important characteristics of the available information regarding the immune response against SARS-CoV-2 virus are revised briefly. In addition, this review summarizes the knowledge on the cellular and molecular mechanisms involved in the improvement of respiratory antiviral defenses by beneficial microorganisms such as *Lactobacillus rhamnosus* CRL1505. The ability of orally administered beneficial microorganisms to differentially modulate the innate and adaptive immune responses in the respiratory tract and systemically clearly indicate the potential of immunobiotics to favorably influence the immune response against SARS-CoV-2 virus.

## Relevant Features of the Immune Responses to SARS-CoV-2

In this section, we summarize the principal findings of SARS-CoV-2 infection and the extrapolations to SARS-CoV and MERS-CoV that were made by scientists in an attempt to establish a definitive view of both the protective immune response and the immunopathology in COVID-19. We highlight the immunological mechanisms involved in the response to SARS-CoV-2 infection that could be potentially modulated by nutritional immunobiotic interventions.

### Respiratory Innate Antiviral Immune Response and SARS-CoV-2

The first barrier involved in the protection of the respiratory mucosa form viral attacks is the respiratory epithelium through its capacity to produce a mucus layer that is in constant displacement because of the action the ciliary movement. The viral particles that reach the mucus layer in the conducting airways can be trapped and swept into the throat and eventually removed through the gastrointestinal tract. When respiratory viruses successfully overcome this first barrier, they interact with respiratory epithelial cells from the conducting airways and/or the deep lung, starting their attachment and internalization to initiate their replication (Hammad and Lambrecht, 2011). Those respiratory epithelial cells as well as antigen presenting cells including dendritic cells (DCs) from the airways and the lung parenchyma, and alveolar macrophages are the first to encounter viruses that enter the respiratory tract. During the viral replication, several pathogen-associated molecular patterns (PAMPs) are exposed, which are recognized by pattern-recognition receptors (PRRs) expressed in respiratory epithelial cells, DCs and alveolar macrophages (Neyt and Lambrecht, 2013; Garbi and Lambrecht, 2017). The PAMPs-PPRs interaction leads to the activation of several signaling pathways that induce the production of type I and II interferon (IFNs) and inflammatory cytokines. IFNs such as IFN-β produced by virus-infected cells are able to act on neighboring immune and non-immune cells promoting an antiviral state through the up-regulation of hundreds of interferon-stimulated genes (ISGs) that counteract viral replication. On the other hand, inflammatory cytokines such as TNF-α, IL-1β, IL-6, IL-8, MCP-1, and IL-17 among others induce the recruitment and activation of immune cells (NK cells, neutrophils, and macrophages) to promote the elimination of virus-infected cells (Hammad and Lambrecht, 2011; Neyt and Lambrecht, 2013; Garbi and Lambrecht, 2017). These defense mechanisms of the innate antiviral immunity are important because they are in several cases sufficient to induce the complete elimination of the respiratory virus or instead, to support the generation of efficient cellular and humoral adaptive immune responses.

The proper and timely production of type I IFNs in the respiratory tract in response to virus infection is crucial to suppress viral replication and dissemination at an early stage. It was shown that coronaviruses are particularly adapted to evade immune detection and dampen this initial antiviral response mediated by type I IFNs (Lessler et al., 2009; Kindler et al., 2016; Channappanavar and Perlman, 2017). An active suppression of IFNs-mediated antiviral response was described for both SARS-CoV and MERS-CoV. These two coronaviruses use multiple strategies to obstruct the type I IFN production and the signaling downstream of the IFNAR. SARS-CoV and MERS-CoV are able to modify the ubiquitination and the degradation of the adaptor molecules mitochondrial antiviral-signaling proteins (MAVs), TNF receptor-associated factor 3 (TRAF3) and TRAF6 impairing type I IFN induction (Kindler et al., 2016). In addition, both coronaviruses are capable of inhibiting interferon regulatory factor 3 (IRF3) nuclear translocation (Kindler et al., 2016). The strength of the interference with the signaling leading to type I IFN production was associated with the disease severity (Channappanavar and Perlman, 2017). This mouse model of SARS-CoV infection demonstrated that the deregulated type I IFN production was the main cause of lethal pneumonia and that inflammatory phagocytes actively participated in the inflammatory damage. The delayed type I IFN production in response to SARS-CoV or MERS-CoV infection compromises the early viral control, leading to influx of hyperinflammatory neutrophils and monocytes-macrophages, which contribute to lung immunopathology through the production of inflammatory factors (Channappanavar and Perlman, 2017). The same work demonstrated that exogenous type I IFNs have both beneficial and detrimental effects depending on the time of administration. Early administration of IFNs contributed to protection against coronavirus infection while delayed IFNs treatment potentiated inflammatory-damage. Interestingly, the efficient impairment of the earlier type I IFNs response by coronaviruses was proposed to explain why SARS-CoV and MERS-CoV tend to have a longer incubation period (up to 11 days) when compared to other respiratory viruses (up to 4 days for IFV) (Lessler et al., 2009). It has been demonstrated that for SARS-CoV-2 the response to viral infection by type I IFN is suppressed (Rokni et al., 2020) and therefore, similar to other respiratory coronaviruses the degree of immune interference would be involved in both the lung immunopathology and the extended incubation period. Moreover, the delayed early activation of the antiviral innate immune response has been proposed to explain the SARS-CoV-2 transmission from asymptomatic infected individuals (Prompetchara et al., 2020). Importantly, recent *in vitro* studies showed that SARS-CoV-2 is sensitive to the treatment with IFN-α or IFN-β demonstrating the potential efficacy of human type I IFNs therapy for suppressing SARS-CoV-2 infection (Mantlo et al., 2020).

### Respiratory Adaptive Antiviral Immune Response and SARS-CoV-2

Research of the adaptive immune responses against respiratory coronaviruses strongly indicated that appropriate Th1 and humoral immune responses are needed for the successful control of SARS-CoV and MERS-CoV infections (Prompetchara et al., 2020) and probably also for SARS-CoV-2. Studies of T cell responses in patients infected with SARS-CoV showed that virus-specific CD8^+^ T cells were more frequent than CD4^+^ T cells (Li et al., 2008). Moreover, those virus-specific T cells had a polyfunctional phenotype with CD4^+^ T cells producing IFN-γ, TNF-α, and IL-2 and CD8^+^ T cells producing IFN-γ and TNF-α and having a degranulated state. Of note, patients resolving the infection had a strong Th1 response that correlated with higher neutralizing antibodies production. In contrast, fatal cases of SARS-CoV infection showed prevalence of Th2 cytokines (IL-4, IL-5, IL-10) in serum together with low production of neutralizing antibodies (Li et al., 2008). Interestingly, experiments with MERS-CoV demonstrated the ability of the virus to down-regulate the expression of MHC-I and MHC-II molecules in macrophages and DCs, impairing antigen presentation, and diminishing T cells activation (Shokri et al., 2019). Epigenetic analysis suggested that DNA methylation plays a crucial role in MERS-CoV-mediated antagonism of antigen-presentation gene expression (Menachery et al., 2018). Although these molecular mechanisms have not been investigated in detail for SARS-CoV-2, clinical studies conducted since the beginning of the COVID-19 pandemic have made clear the ability of this virus to affect the adaptive immunity (Baruah and Bose, 2020; Li G. et al., 2020; Rokni et al., 2020). A transcriptomic study evaluating the expression of immune genes in patients suffering from COVID-19 found significantly reduced levels of *HLA-DRB1, HLA-DMA, HLA-DMB, IL23A*, and *CD74* in severe cases indicating an impairment of the adaptive immune response (Ong et al., 2020).

A common finding in the laboratory analysis of patients infected with SARS-CoV-2 is the simultaneous neutropenia and lymphopenia (Chen X. et al., 2020; Huang et al., 2020; Qin et al., 2020; Shi et al., 2020; Xu et al., 2020; Zhang B. et al., 2020a; Zheng et al., 2020). In fact, the increase in the neutrophil-to-lymphocyte ratio usually indicates higher disease severity and poor clinical outcome. Reduced numbers of total T cells, B cells, and NK cells are consistently observed in patients suffering COVID-19 (Huang et al., 2020; Qin et al., 2020; Shi et al., 2020; Xu et al., 2020). Decreased numbers of CD4^+^ and CD8^+^ T cells as well as increased expression of exhaustion markers such as NKG2A in NK cells and CD8^+^ T cells have been detected in patients infected with SARS-CoV-2 (Chen X. et al., 2020; Zheng et al., 2020). In addition, the strong decline of CD3^+^CD4^+^IFN-γ^+^ T cells and the tendency in the reduction of regulatory T cells was more pronounced in severe cases (Liu et al., 2020; Pedersen and Ho, 2020; Qin et al., 2020).

On the other hand, the humoral immune response, especially the production of neutralizing antibodies, had a protective role against respiratory coronavirus infections. Neutralizing antibodies are capable of limiting the infection at a later phase and prevent reinfection in the future (Gorse et al., 2020). Studies in patients recovered from SARS-CoV infection demonstrated that almost 90% of them had virus-specific IgG neutralizing antibodies after 2 years of the infection (Hsueh et al., 2004; Liu et al., 2006). In contrast, it was shown an antibody-dependent enhancement of virus infection for the MERS-CoV (Wan et al., 2020). In fact, a neutralizing monoclonal antibody targeting the receptor-binding domain of the MERS-CoV S protein can enhance viral entry (Wan et al., 2020). Large-scale single-cell RNA sequencing of B cells isolated from convalescent patients of SARS-CoV-2 infections demonstrated the presence of SARS-CoV-2 spike glycoprotein specific neutralizing antibodies (Shen et al., 2020). It was reported that patients with severe disease frequently had an increased IgG response and a higher titer of total antibodies, which was associated with worse outcome (Zhang B. et al., 2020b; Zhao et al., 2020). On the contrary, the convalescent plasma containing neutralizing antibodies was used successfully to improve the outcome of critically ill patients infected with SARS-CoV-2 who developed ARDS (Shen et al., 2020). Interestingly, a clinical study that investigated the characteristics of the humoral immune response in SARS-CoV-2-infected and uninfected children found that there was rapid production of protective antibodies after first virus exposure (Zhang Y. et al., 2020b). Moreover, the work reported that most of the SARS-CoV-2 specific IgM switched to IgG within 1 week and suggested that this efficient humoral immune response should be responsible of the milder symptoms of SARS-CoV-2-infected children when compared to adults. These findings indicate that the type and concentration of antibodies produced in response to respiratory coronaviruses are able to affect positively or negatively the resolution of the infection.

### Respiratory Inflammatory Response and SARS-CoV-2

Highly pathogenic coronaviruses such as SARS-CoV or MERS-CoV predominantly infect the lower respiratory tract where they rapidly replicate inducing massive inflammatory cell infiltration and elevated pro-inflammatory cytokine and chemokine responses resulting in lung injury (Channappanavar and Perlman, 2017; Merad and Martin, 2020; Tay et al., 2020). In SARS-CoV-infected patients with severe disease, there are high serum levels of pro-inflammatory cytokines such as IL-1β, IL-6, IL-12, and IFN-γ as well as chemokines including IL-8, CCL2, CXCL9, and CXCL10 when compared to patients with uncomplicated SARS (Channappanavar and Perlman, 2017). This so-called “cytokine storm” actively participates in the inflammatory-mediated lung injury and viral sepsis as well as in other complications including pneumonitis, ARDS, respiratory failure, shock, and organ failure. In line with these previous findings for SARS-CoV or MERS-CoV (Nicholls et al., 2003; Wong et al., 2004; Mahallawi et al., 2018), the presence of both lymphopenia and cytokine storm seems to play a critical role in the pathogenesis of SARS-CoV-2 (Merad and Martin, 2020; Tay et al., 2020). A study of severe cases of COVID-19 hospitalized patients demonstrated high levels of pro-inflammatory cytokines in serum samples including IL-2, IL-7, IL-10, G-CSF, CCL2, CCL3, CCL4, CXCL10, and TNF-α (Huang et al., 2020). Those finding were corroborated by several subsequent studies (Chen C. et al., 2020; Qin et al., 2020). The transcriptional profile of whole blood samples from severe cases of SARS-CoV-2 infection revealed that the most up-regulated genes clustered in the “Toll-like receptor (TLR) and inflammatory response” and the “Cytokine signaling” pathways (Ong et al., 2020). The study also concluded that expression of *IL1A* and *IL1B* preceded the nadir of respiratory function (Ong et al., 2020). In addition, it was reported that the peripheral blood of patients with severe COVID-19 contained a significant proportion of CD14^+^CD16^+^ monocytes producing IL-6, highlighting the role of this immune cell population in the hyper-inflammation induced by SARS-CoV-2 infection (Wen et al., 2020; Zhang Y. et al., 2020a).

Recent studies evaluating the broncho-alveolar fluid of patients with severe SARS-CoV-2 infection demonstrated elevated levels of the chemokines CCL2 and CCL7, which are known to be potent chemotractant for the recruitment of CCR2^+^ monocytes (Zhou et al., 2020). Moreover, single-cell RNA sequencing analysis of cells from broncho-alveolar lavages collected from patients with mild or severe COVID-19 indicated that the former group had significantly higher levels of inflammatory monocytes while the resident alveolar macrophage population was diminished (Liao et al., 2020). Therefore, the un-regulated production of pro-inflammatory cytokines during SARS-CoV-2 infection may lead to a massive infiltration of neutrophils and macrophages into the lungs promoting alveolar damage and diffuse thickening of the alveolar wall. The cytokine storm promoted by SARS-CoV-2 may also participate in the tissue damage in the heart, liver and kidney, as well as in spleen atrophy and lymph node necrosis observed in deceased patients (Cao, 2020).

### Coagulation Activation and SARS-CoV-2

In addition to changes in white blood cell populations and cytokine levels, laboratory analyzes of patients infected with SARS-CoV-2 often show thrombocytopenia (Yin et al., 2020; Zhang B. et al., 2020a), alterations of prothrombin time and increases in the levels of D-dimers and fibrinogen degradation products (FDPs) (Huang et al., 2020; Yin et al., 2020; Zhang D. et al., 2020b). Elevation in D-dimers is reflective of increased thrombosis risk that may lead to acute coronary syndrome in COVID-19 patients (Guan et al., 2020; Huang et al., 2020; Wang D. et al., 2020). A study in which critical SARS-CoV-2-infeceted patients were evaluated during their attention in the intensive care unit reported elevated D-dimers and increases in FDPs (Zhang D. et al., 2020b). Another clinical study compared hemostatic parameters between groups of SARS-CoV-2-inefected patients that survived or not to the infection. In non-survivors, higher D-dimers and FDPs levels, longer prothrombin time and activated partial thromboplastin time were observed when compared to the survivors group (Tang et al., 2020). In both studies, disseminated intravascular coagulation was observed in fatal cases. Han et al. (2020) also reported differences in the levels of D-dimers, fibrinogen, and FDPs when patients with milder forms of SARS-CoV-2 infection were compared with the severe cases. Interestingly, the work also demonstrated diminished values of antithrombin in COVID-19 patients than in healthy controls.

It should be noted that the hemostatic alterations were more notable in patients requiring intensive care unit assistance (Guan et al., 2020; Huang et al., 2020; Wang D. et al., 2020). In fact, abnormal coagulation results were associated with poor prognosis of COVID-19 and the existence of disseminated intravascular coagulation was common in deaths associated with this infectious disease. In the points of lung injury mediated by viral replication and cytokine release, there is an activation of monocytes and endothelial cells that increase their secretion of tissue factor (TF) and von Willebrand factor. These hemostatic changes results in the circulation of free thrombin, activation of platelets and stimulation of fibrinolysis creating a pro-thrombotic state and secondary hyperfibrinolysis condition. The disseminated intravascular coagulation mediates pulmonary congestion with microvascular thrombosis as well as other thrombosis and vascular occlusive events that are observed in critically ill COVID-19 patients (Wang J. et al., 2020).

### Intestinal Infection and SARS-CoV-2

It was demonstrated that there is a ubiquitous expression of ACE2, the viral receptor for SARS-CoV-2, in the luminal surface of the gastrointestinal tract, particularly in enterocytes. This fact has made scientists to wonder if the gastrointestinal mucosa could serve as a secondary site for SARS-CoV-2 infection (Gheblawi et al., 2020; Wong et al., 2020). In support of this hypothesis, it was shown that 5 to 10% of the patients infected with SARS-CoV-2 developed intestinal symptoms such as diarrhea, vomiting, and abdominal pain (Guo et al., 2020; Ji et al., 2020; Li L. et al., 2020; Song et al., 2020; Wang D. et al., 2020; Yeo et al., 2020). Despite the fact that the percentage of SARS-CoV-2-infecetd patients with intestinal symptoms is less than that found for SARS-CoV or MERS-CoV (20–25%) (Wong et al., 2020), those studies suggest that SARS-CoV-2 can actively infect and replicate in the gastrointestinal tract. The possibility that the virus may replicate efficiently in the intestinal tract, opens other questions related to both its pathogenesis and its transmission. Studies in experimental animals in which the ACE2 was disrupted, demonstrated that gut dysbiosis is a prevalent finding (Cole-Jeffrey et al., 2015; Oliveira et al., 2020). Moreover, the intestinal microbial dysbiosis associated to ACE2 alterations was associated to exacerbations of diabetes, hypertension, and pulmonary diseases (Cole-Jeffrey et al., 2015; Oliveira et al., 2020). In line with these findings in animal models, studies performed during SARS-CoV outbreak in the early 2000s demonstrated that patients with viral shedding from the gastrointestinal tract had a more aggressive clinical course (Leung et al., 2003; Cheng et al., 2004). Some evidence also support that SARS-CoV-2 may induce alterations of intestine-blood barrier promoting the abnormal absorption of microbial molecules or the spread of bacteria that strongly stimulate the systemic inflammatory response contributing to multiorgan dysfunction and septic shock (Guan et al., 2020; Huang et al., 2020; Wang D. et al., 2020). On the other hand, the replication of the virus in the intestinal tract highlight the importance of fecal-oral route in SARS-CoV-2 transmission, which could be of relevance in certain circumstances such as crowded conditions of large family groups (Yuen et al., 2020). This has important implications to the disease management, transmission, and infection control.

## Modulation of Respiratory Immunity by the Intestinal Microbiota

**Figure 2:**
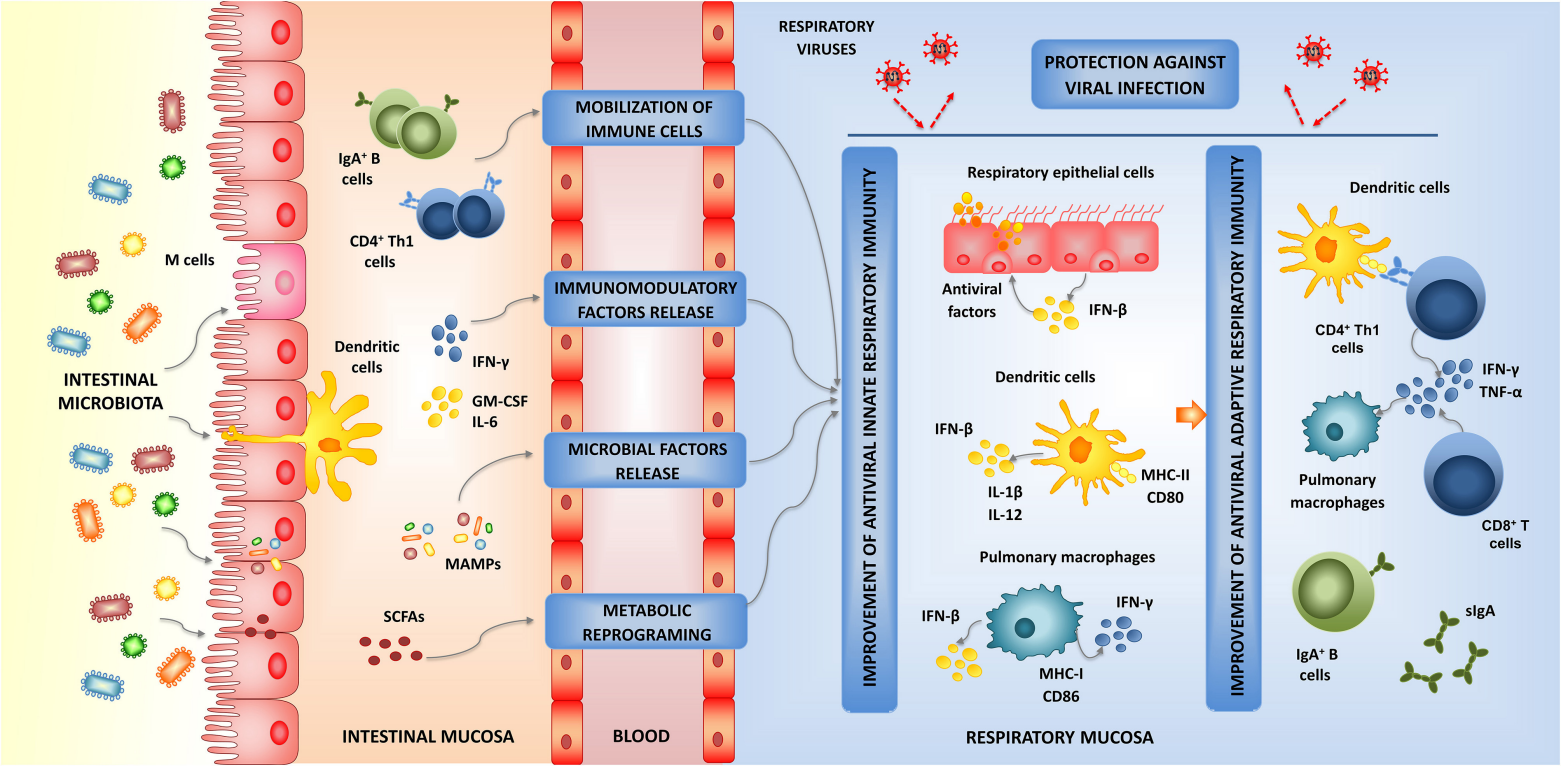
Modulation of respiratory antiviral immunity by intestinal microbiota. Proposed mechanism for the distal immunomodulation induced by the intestinal microbiota and the enhancement of the resistance against viral infections through the improvement of the respiratory innate and adaptive antiviral immune responses.

The effect of the intestinal microbiota on the immune responses in distal mucosal sites and its impact on the outcome of respiratory infections has been explored recently. In this regard, some studies reported an important role for intestinal microbiota in maintaining respiratory antiviral immunity through the modulation of the immune response both at the steady state as well as in response to the viral attack (Ichinohe et al., 2011; Abt et al., 2012; Bradley et al., 2019). Remarkably, most studies demonstrated a significant impact of the intestinal microbiota in the respiratory innate antiviral defense mechanisms thought its influence on (a) respiratory epithelial cells, (b) respiratory DCs, and (c) pulmonary macrophages. In addition, the influence of the intestinal microbiota on the antiviral innate immunity also modifies the respiratory antiviral cellular and humoral adaptive immune responses (Figure 2). (a) The influence of intestinal microbiota on the antiviral immune response mediated by respiratory epithelial cells was demonstrated by bone marrow chimera experiments, which identified non-immune respiratory cells as crucially important for early antiviral immunity and their interaction with immune cells important for late antiviral resistance (Bradley et al., 2019). It was shown that the intestinal microbiota influence the IFN-α/β receptor (IFNAR) surface expression in respiratory epithelial cells, which in front of a respiratory virus attack are able to respond more efficiently to type I IFNs stimulation with enhanced ISGs levels. Thus, the microbiota-driven IFN signature in the respiratory epithelia impedes early virus replication (Bradley et al., 2019). (b) The influence of the intestinal microbiota on the antiviral immune response mediated by DCs was reported by Ichinohe et al. (2011). It was demonstrated that the intestinal microbiota, in particular Gram positive bacterial populations, are involved in the appropriate distribution and activation of respiratory DCs at steady state. Intact intestinal microbiota support the expression of pro–IL-1β, pro–IL-18, and NLRP3 at steady state, and this inflammasome activation improve the activation and migration of DCs from the lung to the draining lymph node after the challenge with IFV. This effect of the intestinal microbiota was of importance for the efficient activation of virus specific CD8^+^ and CD4^+^ T cells, the expansion of B cells and the production of virus-specific antibodies (Ichinohe et al., 2011). (c) The effect of the intestinal microbiota on the antiviral immune response mediated by macrophages was studied by Abt et al. (2012). It was also shown that the intestinal microbiota help to maintain the optimal functions of pulmonary macrophages. The intestinal microbiota is involved in the efficient capacity of pulmonary macrophages to produce type I IFNs and antiviral ISGs including *Irf7, Mx1*, and *Oas1* to limit viral replication (Abt et al., 2012). In addition, the intestinal microbiota positively influence the ability of pulmonary macrophages to support the generation of virus-specific antibodies as well as virus-specific T cells. In intestinal microbiota-depleted mice, macrophages have a reduced expression of MHC-I and CD86 molecules. An impaired number of IFV-specific CD8^+^ T cells as well as a reduced ability of these cells to produce IFN-γ, TNF-α, IL-2, and MIP-1α was also observed (Abt et al., 2012).

Those studies demonstrated that the signals provided by the intestinal microbiota act at multiple levels in the respiratory mucosa stimulating an antiviral state in non-immune cells and innate immune cells that would allow an efficient control of viral replication early during the infection. The improved respiratory innate antiviral immune response would enhance the functionality of immune cells leading to improved humoral and cellular adaptive immune responses later in the course of viral infection (Figure 2). Although these effects of the intestinal microbiota have been documented for IFV infection (Ichinohe et al., 2011; Abt et al., 2012; Bradley et al., 2019), it should be considered that the cellular and molecular mechanisms involved in the innate antiviral immune response are not virus specific and therefore, they are similar for all the respiratory viruses. Then, it can be assumed that the beneficial microbes in the intestinal tract may favorably influence the innate immune responses to other respiratory viruses as well. In this regard, it was recently demonstrated that a high-fiber diet improved the production of acetate by the intestinal microbiota of mice and that this metabolic product modulated the activity of respiratory IFN-β and increased the expression of ISGs in the lung (Antunes et al., 2019). The immunological changes induced in the respiratory tract by the dietary treatment significantly increased the resistance of mice to the challenge with respiratory syncytial virus (RSV).

The exact origin and nature of the signals used by the intestinal microbiota to modulate the respiratory antiviral immunity remain to be determined. Four probable mechanisms have been proposed, which are not mutually exclusive, to explain the effect of the intestinal microbiota on the respiratory immunity (Figure 2). The existence of the so-called common mucosal immune system implies that the immune cells activated in one mucosal tissue can mobilize and reach distant mucosal sites where they can influence immune responses. Then, the mobilization of B and T cells from the intestinal mucosa to the respiratory tract could be involved in the beneficial effects exerted by the intestinal microbiota (Kitazawa and Villena, 2014; Zelaya et al., 2016). It was also hypothesized that immune factors such as cytokines and growth factors produced in the intestinal mucosa in response to microbiota stimulation, can be released to blood and act systemically or in other mucosal tissues (Kitazawa and Villena, 2014; Zhang D. et al., 2020a). There is also evidence that microbial products can be absorbed in the intestine and circulate throughout the host (Macpherson and Uhr, 2004; Clarke et al., 2010). It is speculated that some microbial-associated molecular patterns (MAMPs) derived from the intestinal microbiota can be adsorbed and transported to extraintestinal sites where they stimulate PRRs expressed in non-immune and immune cells influencing the immune responses. Perhaps the best example of this mechanism was given by Clarke et al. (2010) who demonstrated that the peptidoglycan translocated from the gut microbiota to the systemic circulation was detected by NOD1, resulting in enhanced systemic innate immunity mediated by neutrophils. In addition, microbial metabolites that are adsorbed in the intestine have been associated to differential modulation of respiratory immune responses. This effect has been called “metabolic reprograming” and implies that metabolites such as circulating short-chain fatty acids (SCFAs) (Trompette et al., 2014), desaminotyrosine (Steed et al., 2017), and docosahexanoic acid (Fonseca et al., 2017) can reach innate immune cells in the respiratory tract and improve their responses to viral infections.

Interestingly, the studies with germ-free as well as antibiotic-treated mice recolonized with bacteria demonstrated that the changes induced by the intestinal microbiota are reversible and more importantly, tunable (Ichinohe et al., 2011; Abt et al., 2012; Steed et al., 2017; Bradley et al., 2019). Moreover, it was shown that not all commensal bacteria could contribute equally to the immunocompetence in the lung (Ichinohe et al., 2011). These findings have opened the possibility of exploring particular strains of beneficial bacteria with immunomodulatory capacities, referred to as immunobiotics, in order to increase antiviral defenses in the respiratory tract. Then, several immunomodulatory beneficial microbial strains, mainly form the *Lactobacillus* and *Bifidobacterium* species, have been tested in their capacities to modulate the respiratory antiviral immune response when orally administered (reviewed in Zelaya et al., 2016; Villena et al., 2019).

## Modulation of Human Respiratory Antiviral Immunity by Immunobiotics

A growing number of studies have examined the effect of immunobiotic nutritional interventions on the incidence, the duration and severity of respiratory infections in humans. Several clinical trials, systematic reviews and meta-analyses have suggested that immunobiotics may be effective in improving the resistance of children, adults, and even the elderly against respiratory infections such as the common cold and influenza-like symptoms. A meta-analysis of four clinical trials in which 1,800 children participated demonstrated that *L. rhamnosus* GG administration reduced incidence of acute otitis media, the risk of upper respiratory infections and the days of antibiotic treatments (Liu et al., 2013). Similarly, a meta-analysis of 23 double-blinded, randomized, and placebo-controlled trials involving more than 6,000 children demonstrated that immunobiotic treatments are capable of reducing the numbers of respiratory tract infections cases, days of illness per person, and days absent from school compared to placebo groups (Wang et al., 2016). Interestingly, immunobiotic intervention was shown to reduce the episodes of viral respiratory infections even in asthmatic children (Ahanchian et al., 2016). The improvement of respiratory defenses were also demonstrated in healthy adults. The nutritional intervention with the well-know immunobiotic strain *L. casei* Shirota was capable of diminishing the cumulative number of respiratory infectious episodes and the cumulative days with symptoms per person (Shida et al., 2017). An increase in blood leukocyte, neutrophil, and NK cell counts and activity was found in shift workers treated with *L. casei* DN-114001. The work also registered a significant reduction in the incidence of respiratory infections in DN-114001-treated adults when compared to the placebo group (Guillemard et al., 2010a). Moreover, the same probiotic treatment was found to diminish the incidence and severity of upper respiratory tract infections in the elderly (Guillemard et al., 2010b). Middle-aged and elderly people treated with a probiotic yogurt containing *L. paracasei* N1115 have a reduced incidence of acute upper respiratory tract infections and higher blood CD3^+^ T cells levels were associated to the beneficial effect (Pu et al., 2017). Two independent studies conducted in healthy elderly individuals demonstrated that the administration of *L. delbrueckii* ROLL1073R-1 augmented NK cells activity and reduced the risk of catching the common cold (Makino et al., 2010).

Although the clinical studies demonstrated the beneficial effect of immunobiotics on human respiratory infections, few of them investigated specific viral etiology from the respiratory tract, and reported a significant decrease in viral load (Lehtoranta et al., 2014). On the other hand, the impact of immunobiotics on the human respiratory and systemic antiviral immune responses have been clearly demonstrated in several studies in which the immunobiotic interventions acted as efficient adjuvants on IFV vaccination (reviewed in Zelaya et al., 2016).

Collectively, research has shown that some immunobiotic strains are capable of making a difference in the host's response to respiratory viral infections. However, there are variable findings in the effect of immunobiotics on respiratory tract infections in humans. This fact could be related to several factors including the specific strain, the duration of regimens, administration forms, doses, and follow-up time. Therefore, the role of immunobiotics on specific viruses needs deeper research with randomized, double-blind, and placebo-controlled trials in different age populations. Moreover, the cellular and molecular mechanisms involved in the beneficial effects of each particular strain have not been extensively evaluated. Those kind of studies are necessary to provide solid scientific basis to promote the use of immunobiotics in the prevention of respiratory viral infections.

**Figure 3:**
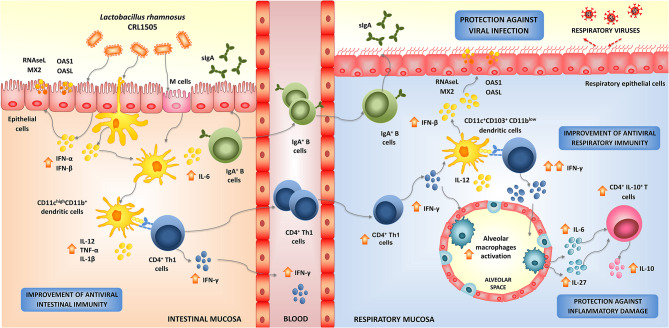
Modulation of respiratory antiviral immunity by *Lactobacillus rhamnosus* CRL1505. Proposed mechanism for the distal immunomodulation induced by the immunobiotic strain *L. rhamnosus* CRL1505 and the enhancement of the resistance against viral infections through the improvement of the respiratory innate and adaptive antiviral immune responses.

In a randomized controlled trial in children, it was demonstrated that the immunobiotic strain *L. rhamnosus* CRL1505, administered in a yogurt, improved mucosal immunity and reduced the incidence and severity of viral intestinal and respiratory infections (Villena et al., 2012b). Since then and for more than a decade, we have dedicated our efforts to perform detailed mechanistic studies by using *in vivo, in vitro*, and *in silico* models to characterize the antiviral properties of *L. rhamnosus* CRL1505 (Figure 3). The most relevant findings of *L. rhamnosus* CRL1505's impact on mucosal antiviral immunity are summarized below.

## Modulation of Mucosal Antiviral Immunity by *L. rhamnosus* CRL1505

### Respiratory Innate Antiviral Immune Response and *L. rhamnosus* CRL1505

In order to study in depth the mechanisms involved in the enhancement of respiratory antiviral immunity mediated by *L. rhamnosus* CRL1505, we conducted studies in animal models using two respiratory viruses of great importance in public health: RSV and IFV (Chiba et al., 2013; Zelaya et al., 2014). Our results showed that orally administered CRL1505 strain was capable of significantly reducing viral titers in lungs, lessened pulmonary damage, and increased survival of mice in response to RSV (Chiba et al., 2013) or IFV challenges (Zelaya et al., 2014). The protective effect induced by *L. rhamnosus* CRL1505 was associated its ability to differentially modulate the innate antiviral immune response, particularly to its capacity to improve the levels of type I IFNs in the respiratory tract. Mice treated with the immunobiotic strain had significantly higher levels of IFN-β in the respiratory mucosa after the nasal challenge with TLR3 agonist, RSV, or IFV (Villena et al., 2012a; Chiba et al., 2013; Zelaya et al., 2014). Type I IFNs binding to their cognate cell surface receptors activate positive feedback loops that amplifies the expression of IFNs as well as hundreds of different ISGs. This IFNs release then efficiently amplifies the expression of antiviral proteins targeting a variety of viral replication steps in uninfected bystander cells. Considering that, *L. rhamnosus* CRL1505 was able to induce and earlier and higher production of IFN-β, it can be speculated that the generation of a fast and effective antiviral state in the respiratory tract could be involved the higher resistance against viral infections. Our group is still investigating how the orally administered CRL1505 strain can modulate the expression of type I IFNs and antiviral factors in the respiratory tract. We have demonstrated increased numbers of CD4^+^IFN-γ^+^ T in the intestine of mice orally treated with *L. rhamnosus* CRL1505, and a mobilization of these cells from the gut to the respiratory mucosa (Villena et al., 2012a; Chiba et al., 2013). Then, the improved production of IFN-γ and the subsequent stimulation DCs and macrophages in the lungs could be involved in the enhanced anti-viral state induced by *L. rhamnosus* CRL1505 (Figure 3). In line with this hypothesis, we have recently demonstrated that orally administered CRL1505 strain is able to increase the production of IFN-β in CD11c^+^SiglecF^+^ alveolar macrophages, which is further augmented in response to TLR3, RSV, or IFV challenges (*submitted for publication*). Since the activation of the innate antiviral immune response is not virus specific, it is tempting to speculate that *L. rhamnosus* CRL1505 would improve the resistance to other respiratory viruses as well (Figure 3).

### Respiratory Adaptive Antiviral Immune Response and *L. rhamnosus* CRL1505

As mentioned above, orally administered CRL1505 strain induced the mobilization CD3^+^CD4^+^IFN-γ^+^ T cells from the gut to the respiratory tract, increasing the local production of IFN-γ and stimulating antigen presenting cells (Villena et al., 2012a; Chiba et al., 2013; Zelaya et al., 2014). When lung DCs populations were evaluated in mice after the oral treatment with *L. rhamnosus* CRL1505 it was found increased levels of both CD11c^+^CD103^+^ and CD11c^+^CD11b^high^ DCs. In addition, both DCs populations showed higher expression of MHC-II when compared with controls (Villena et al., 2012a; Chiba et al., 2013). Moreover, IL-12 and IFN-γ were increased specially in CD11c^+^CD103^+^ DCs, the antigen presenting cell population that has been associated to the generation of Th1 responses in the respiratory tract (Kitazawa and Villena, 2014). The stimulation of respiratory DCs further increased the local numbers of CD3^+^CD4^+^IFN-γ^+^ T cells later in the course of RSV or IFV infections (Figure 3; Villena et al., 2012a; Chiba et al., 2013).

The improvement in antigen presentation would be associated not only with the higher levels of IFN-γ in the respiratory tract but also with the enhanced levels of type I IFNs induced by the immunobiotic treatment (Villena et al., 2012a; Chiba et al., 2013). It was reported that DCs produce IFN-α and IFN-β and undergo maturation in response to the paracrine and autocrine actions of type I IFNs. Moreover, both IFN-α/β have been shown to be a crucial link between innate and adaptive immunity because of their ability to stimulate DCs responses *in vivo* (Le Bon and Tough, 2002). The treatment of DCs with type I IFNs activate these antigen presenting cells enhancing their expressions of MHC-II, CD40, and CD86 molecules, and improving their capacity to initiate CD4^+^ and CD8^+^ T cells responses (Montoya et al., 2002; Yoo et al., 2010). Of note, we were not able to detect improved numbers of CD3^+^CD8^+^IFN-γ^+^ T cells in the respiratory tract of CRL1505-treated mice after the challenges with respiratory viruses (Chiba et al., 2013; Zelaya et al., 2014). Further studies are necessary to find out whether the immunobiotic strain is able to positively influence other functions of CD8^+^ T cells such as their ability to kill virus-infected cells.

On the other hand, the improved antigen presentation mediated by *L. rhamnosus* CRL1505 could also have a beneficial effect on the respiratory humoral immune response. In support of this hypothesis, several other laboratories have demonstrated the ability of orally administered immunobiotics to improve respiratory and systemic humoral immune responses in front of viral infections (reviewed in Zelaya et al., 2016). Moreover, although we have not evaluated the effect of orally administered CRL1505 strain on the respiratory humoral immune response in the context of viral infections, we have demonstrated a beneficial effect on the antibody response to *Streptococcus pneumoniae* infection (Salva et al., 2010). Respiratory pathogen challenges results principally in an IgA response in the airways while their contact with the deep lung induce an increased production of pathogen-specific IgG. Then, both types of antibodies are necessary for the full protection against respiratory pathogens. In our hands, the oral treatment with *L. rhamnosus* CRL1505 significantly increased the levels of anti-pneumococcal respiratory and serum IgA and IgG antibodies (Salva et al., 2010). Considering that the enhancement of the specific antibodies production in the respiratory tract induced by the CRL1505 treatment would be the result of an improved innate immune response and a better antigenic presentation, it is possible to think that the immunobiotic treatment would be also capable to generate a better humoral response against respiratory viruses. In support of this hypothesis, it was demonstrated that type I IFNs promote antibody responses *in vivo* through the stimulation of DCs (Le Bon et al., 2001). It was shown that type I IFNs increase the levels of antigen-specific antibodies of all subclasses of IgG and induced IgG2a and IgG3 antibodies. Furthermore, type I IFNs combined with nasally administered IFV vaccines in immunization protocols have been shown to induce effective humoral immune responses (Bracci et al., 2005).

### Respiratory Inflammatory Response and *L. rhamnosus* CRL1505

TLR3 has a complex role in viral infections since it was reported that this PRR mediated both beneficial and detrimental effects during the course of viral infections such as IFV, RSV, SARS-CoV, or MERS-CoV (Rudd et al., 2005, 2006; Groskreutz et al., 2006; Le Goffic et al., 2006; Totura et al., 2015). It was reported that the inflammatory lesions induced by IFV infection are diminished in the absence of TLR3 (Le Goffic et al., 2006). Knockdown of TLR3 significantly reduced the infiltration of lungs with inflammatory macrophages and CD8^+^ T cells and reduce the production of IL-6, IL-12, and CCL5 in response to IFV infection. On the other hand, it was shown that TLR3 has minimal effect on lung RSV clearance while this PRR actively participates in the respiratory inflammatory damage (Rudd et al., 2005, 2006; Groskreutz et al., 2006). Impaired regulation of TLR3 activation during the course of RSV infection increased the production of inflammatory cytokines and chemokines, promoted the infiltration of inflammatory cells (Rudd et al., 2005; Groskreutz et al., 2006) and potentiated pathogenic Th2-biased response in the lung (Rudd et al., 2006) contributing to respiratory tissue damage and the alteration of lung functionality. More recently, it was also shown that TLR3 signaling pathway also participates in the lung inflammatory damage induced by respiratory coronaviruses infections. It was reported that the knockdown of the TLR3 adaptor TRIF increased the susceptibility of mice to SARS-CoV infection (Totura et al., 2015). Mice lacking TRIF had an aberrant pro-inflammatory cytokine and chemokine response and an increased infiltration of neutrophils that correlated with increased pathology of the lung.

These findings highlight the importance of the appropriate regulation of the TLR3 signaling for generating a balanced protective respiratory innate immune response against viruses. Therefore, we focused our attention in evaluating the effect of orally administered *L. rhamnosus* CRL1505 in the inflammatory immune response triggered in the respiratory tract by TLR3 activation. For this purpose, we utilized the artificial dsRNA analog and TLR3 ligand poly(I:C) to imitate the pro-inflammatory alterations induced by viral infections in the lung. Nasally administered poly(I:C) to infant or adult BALB/c mice increases the levels of pro-inflammatory cytokines and chemokines in the respiratory tract, augments the inflammatory cell recruitment into the lung, induces respiratory epithelial cell death and impairs the alveolar-capillary barrier (Stowell et al., 2009; Aeffner et al., 2011; Villena et al., 2012a). We observed that *L. rhamnosus* CRL1505 treatment induced quantitative and kinetic differences in the production of pro- and anti-inflammatory cytokines in the respiratory tract after TLR3 activation (Villena et al., 2012a). In the first hours after poly(I:C) administration, the levels of respiratory TNF-α and IL-8 in CRL1505-treated mice were similar to controls while IL-6 and CCL2 were significantly higher. Interestingly, 24 h after TLR3 activation, the levels of TNF-α, IL-6, IL-8, and CCL2 were lower in CRL1505-treated mice than in controls. In addition, respiratory IL-10 in *L. rhamnosus* CRL1505-treated mice were significantly increased during all the studied period (Villena et al., 2012a). We have reported that CD3^+^CD4^+^IL-10^+^ T cells are the main source of the immunoregulatory cytokine in the respiratory tract during the course of viral infection (Chiba et al., 2013; Zelaya et al., 2014) and that the improvement of this immune cell population is mediated by the secretion of IL-6 and IL-27 by alveolar macrophages (*submitted for publication*, Figure 3). Then, our results indicated that during the early stages, the production of pro-inflammatory factors would contribute to viral clearance (Chiba et al., 2013; Zelaya et al., 2014). While in the later stages, the reduction of inflammatory cytokines and chemokines together with the improved levels of regulatory cytokines such as IL-10 would be critical for attenuating the inflammatory damage in the lungs induced by TLR3 activation (Villena et al., 2012a), and RSV (Chiba et al., 2013) or IFV infections (Zelaya et al., 2014).

### Coagulation Activation and *L. rhamnosus* CRL1505

In order to evaluate the effect of orally administered *L. rhamnosus* CRL1505 on the activation of coagulation during the course of respiratory viral infection we used poly(I:C) challenge as well as RSV and IFV infections in mice (Zelaya et al., 2014, 2016). The inflammatory response triggered by RSV or IFV infection augment the levels of pro-inflammatory cytokines such as TNF-α, IL-1β, and IL-6 that induce the secretion of TF by endothelial cells and monocytes contributing to pathology and lung tissue injury (Yang and Tang, 2016). In addition, it was reported that the direct stimulation of endothelial cells with TLR3 ligands induce the up-regulation of TF and the down-regulation of thrombomodulin (TM) (Shibamiya et al., 2009). Moreover, *in vivo* studies also demonstrated that administration of poly(I:C) is able to alter vascular permeability and increase the levels of D-dimers indicating that coagulation and fibrinolysis were triggered (Wang et al., 2004). Our own *in vivo* studies were in agreement with these previous reports since we observed that the nasal challenge of BALB/c mice with poly(I:C), RSV, or IFV induced a marked enhancement of lung TF expression and thrombin–antithrombin complex (TATc) levels in the respiratory tract while they reduced lung TM expression. These inflammatory–coagulative modifications were accompanied by respiratory tissue alterations and impairment of lung function (Zelaya et al., 2014, 2016). We also were the first in demonstrating that orally administered CRL1505 strain significantly reduced coagulation activation in blood and in the respiratory tract after the nasal challenge with poly(I:C) or respiratory viruses (Zelaya et al., 2014). Lower levels of respiratory TATc, reduced expression of TF and increased expression of lung TM were found in *L. rhamnosus* CRL1505-treated mice when compared to controls. In addition, by using anti-IL-10R blocking antibodies we demonstrated that IL-10 is important for the regulation of coagulation induced by the immunobiotic CRL1505 strain (Zelaya et al., 2014). IL-10 induced by *L. rhamnosus* CRL1505 administration is able to attenuate the expression of TF in inflammatory macrophages recruited in response to TLR3 activation of viral infection.

### Intestinal Antiviral Immune Response and *L. rhamnosus* CRL1505

We have also advanced in the characterization of the immunomodulatory and antiviral effects of the CRL1505 strain in the intestinal mucosa (Figure 3). *In vitro* studies performed in intestinal epithelial cells showed that *L. rhamnosus* CRL1505 was able to improve the production of type I interferons (Villena et al., 2014). Moreover, transcriptomic studies revealed a remarkable ability of the CRL1505 strain to enhance the expression of IFN-α and IFN-β as well as the antiviral factors *Nplr3, Oas1, OasLA, Mx2*, and *RNAseL* in intestinal epithelial cells when compared to other immunomodulatory lactobacilli (Albarracin et al., 2017, 2020). *In vitro* experiments also demonstrated that *L. rhamnosus* CRL1505 is capable of augmenting the expression of MHC-II and CD80 and the production of IFN-γ in intestinal DCs and macrophages (Villena et al., 2014). The immunological changes induced by the CRL1505 strain in intestinal non-immune and immune cells correlated with the improved resistance of children to rotavirus infection (Villena et al., 2012a). We have also performed *in vivo* studies to evaluate the impact of *L. rhamnosus* CRL1505 on the intestinal inflammatory response triggered by TLR3 activation. Using a murine model of intraperitoneal challenge with poly(I:C) to induce an intestinal inflammatory response, we demonstrate that oral administration of *L. rhamnosus* CRL1505 differentially modulates the intestinal antiviral immune response mediated by TLR3 and intraepithelial lymphocytes (Tada et al., 2016). The beneficial effects of the CRL1505 strain were associated with its ability to decrease the production of inflammatory cytokines including IL-15, increase IL-10 levels and reduce the number of CD3^+^NK1.1^+^ and CD8αα^+^NKG2D^+^ cells in the intestine. Our results indicated that the immunobiotic treatment was capable of reducing damage to intestinal epithelial cells mediated by the TLR3-IL-15-CD8αα^+^NKG2D^+^ cells axis (Tada et al., 2016). Then, we have conclusively demonstrated that a dietary intervention with *L. rhamnosus* CRL1505 is able to increase resistance to challenge with intestinal virus, reducing viral replication and favorably modulating the inflammatory response triggered by the viral attack (Villena et al., 2012a, 2014; Tada et al., 2016; Albarracin et al., 2017, 2020).

## Could Immunobiotics Have Any Benefit in the SARS-CoV-2 Pandemic?

**Figure 4:**
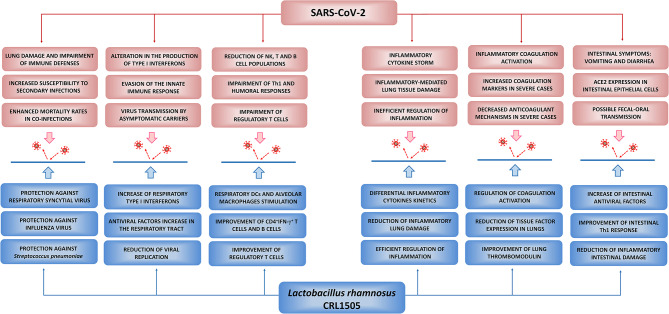
Potential beneficial effects of *Lactobacillus rhamnosus* CRL1505 against SARS-CoV-2 infection. Proposed potential benefits in the reduction of the incidence and severity of COVID-19 induced by nutritional interventions with the immunobiotic strain *L. rhamnosus* CRL1505. The principal pathological and immunological alterations during SARS-CoV-2 infection were summarized from Jamilloux et al. (2020), Tay et al. (2020), Merad and Martin (2020), and Chen N. et al. (2020). The beneficial effects of *L. rhamnosus* CRL1505 in the respiratory tract were summarized from Villena et al. (2012a), Chiba et al. (2013), and Zelaya et al. (2014). The beneficial effects of *L. rhamnosus* CRL1505 in the intestinal tract were summarized from Villena et al. (2014), Tada et al. (2016), and Albarracin et al. (2017, 2020).

As stated before, a clear understanding of the immunological processes underlying the clinical manifestations of COVID-19 is vital for the rational design of effective therapies aimed to prevent or ameliorate the severity of the disease. Here, we provided an overview of the current knowledge of the pathophysiology and the immune response to SARS-CoV-2 infection (Figure 4). In addition, we have summarized the cellular and molecular information gathered during this decade of work regarding the effect of *L. rhamnosus* CRL1505 in the beneficial modulation of the mucosal antiviral immune response. This comparative analysis allows us to postulate that the use of immunobiotic microorganisms such as the CRL1505 strain could be beneficial in the prevention and/or the reduction of the severity of infections caused by emerging respiratory viruses such as SARS-CoV-2 (Figure 4).

**Figure 5:**
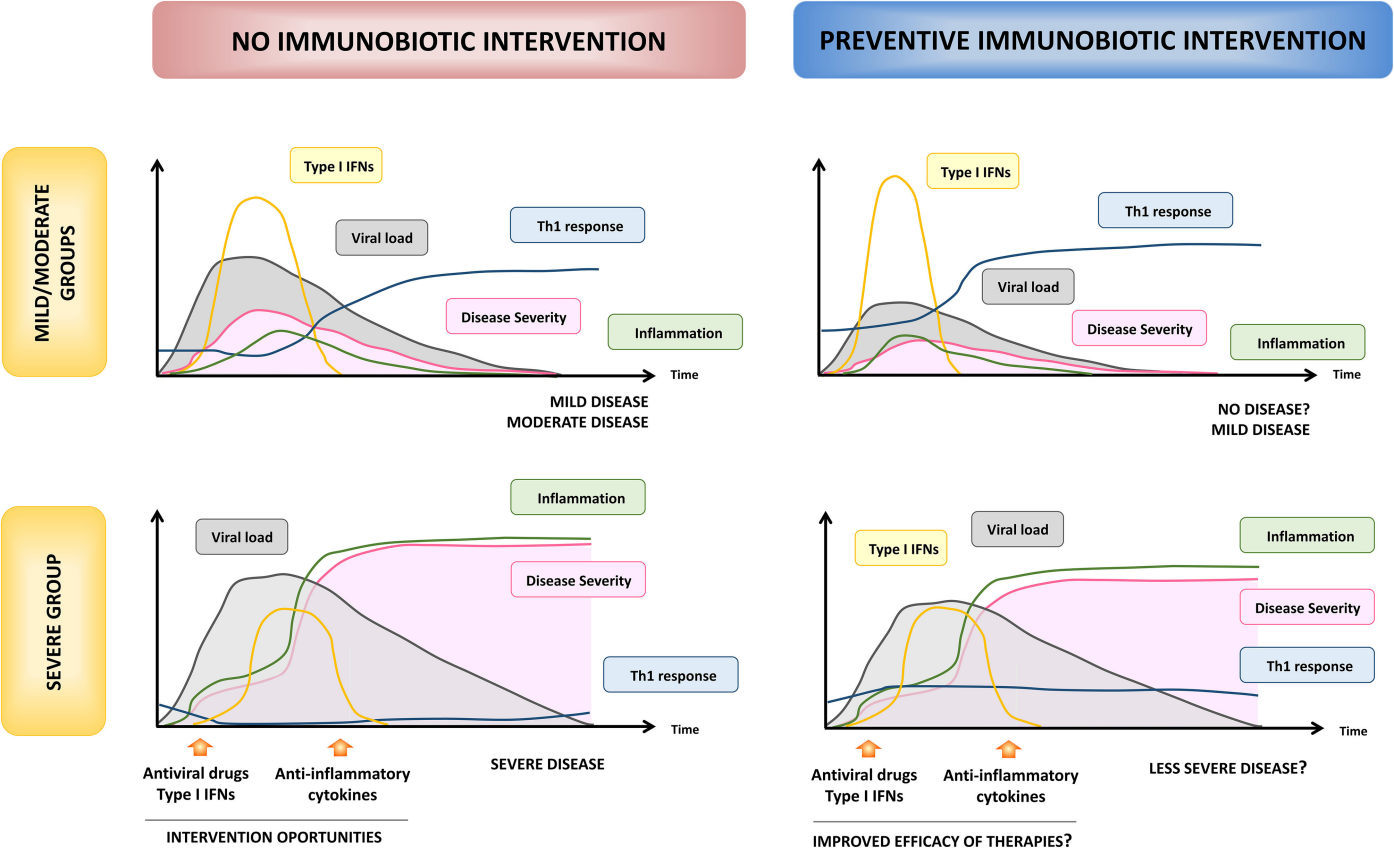
Hypothesis of the beneficial effects of preventive immunobiotic intervention on SARS-CoV-2 infection. The kinetics and intensity of immune actors involved in the response to SARS-CoV-2, viral load and COVID-19 severity are shown according to Jamilloux et al. (2020) in both “mild/moderate” and “severe” susceptible hosts. Optimal times for therapeutic interventions proposed to ameliorate disease severity are also shown (left panels). The potential modulation of the kinetics and intensity of immune response, viral load, and disease severity by preventive nutritional immunobiotic interventions for low and high susceptible hosts are shown in the right panels.

One of the most important questions asked by scientists regarding the efficacy of immunobiotics in reducing infections is whether they can be used as both preventive and therapeutic tools. Data from animal and clinical studies demonstrated that immunobiotic interventions are mostly effective in the prevention of respiratory infections while they rarely influence the course of infections once the pathogen has started its replication in the host (Zelaya et al., 2016; Villena et al., 2019). Thus, in the context of the SARS-CoV-2 pandemic, immunobiotics should be considered as a strategy to strengthen the respiratory immune system of healthy people, rather than as a therapeutic option in patients who have already been infected with the virus. On the other hand, the characteristics of the host in which the immunobiotics are going to be applied would be also determinant for their effectiveness. One of the most important findings made by clinicians and scientists since the COVID-19 pandemic began is the fact that distinct population groups may react differently to SARS-CoV-2 infection. Jamilloux et al. (2020) defined three phenotypes in COVID-19 patients according to the infection's progression and extent: (a) patients with mild and non-specific symptoms who will not progress to severe disease (mild group), (b) patients with localized inflammation, pneumonia and requiring hospitalization (moderate group), and, (c) patients that develop ARDS, systemic hyperinflammation, and multiple organ failure who will require critical care management and are at risk of fatal outcome (severe group). Children, adolescents, and immunocompetent adults are in the mild/moderate group, which represent the 95% of the SARS-CoV-2-infected persons. On the other hand, 5% of COVID-19 patients are adults with comorbidities and the elderly who are at high risk to develop severe disease (Jamilloux et al., 2020). These two groups, “mild/moderate,” and “severe,” greatly differ in the intensity and the kinetics of the immune responses triggered upon SARS-CoV-2 infection (Figure 5). Thus, we can hypothesize that the effectiveness of the nutritional interventions with immunobiotics will depend on the COVID-19 phenotype group considered.

It is tempting to speculate that immunobiotics would have a relevant impact on SARS-CoV-2 infection in the “mild/moderate” group. As highlighted before, this coronavirus is capable of both impair the innate antiviral immune response and induce a hyperactivation of the immune system. However, these two changes in the immune system are not simultaneous (Figure 5) and moreover the latter seems to ensue from the former (Jamilloux et al., 2020; Merad and Martin, 2020; Tay et al., 2020). The efficient viral clearance induced by the activities of type I IFNs in the earlier stages of the infection is a key to prevent the viral dissemination, the T cell exhaustion, and the subsequent hypercytokinemia. Then, the improved type I IFNs response in the early stage of infection would be capable of avoiding viral replication or reducing the severity of the disease. In support of statement, it was suggested that SARS-CoV-2 have a lesser type I IFN antagonism compared to SARS-CoV (Perlman and Netland, 2009) and *in vitro* experiments have demonstrated that SARS-CoV-2 displays a greater sensitivity to type I IFNs than SARS-CoV (Lokugamage et al., 2020). Moreover, mild/moderate infection by SARS-CoV-2 has been associated with a potent type I IFNs response in the respiratory tract (Zhou et al., 2020) and blood (Trouillet-Assant et al., 2020). The efficient management of viral infection in the early stages would also enable the generation of an adequate and timely Th1 response. In fact, improved levels of IFN-γ and Th1 cells in the respiratory tract have been associated to the mild/moderate disease while IFN-γ was not found to be elevated in the sera or respiratory tract of patients with severe forms of COVID-19 (Xiong et al., 2020). Furthermore, lower levels of IFN-γ have been proposed as marker of poorer outcomes (Lagunas-Rangel and Chávez-Valencia, 2020). On the other hand, the rapid control of virus replication would enable the host to be protect against disease progression by limiting epithelial damage, local inflammation, and accumulation of pathological macrophage populations (Jamilloux et al., 2020; Merad and Martin, 2020; Tay et al., 2020).

Considering that immunobiotic strains such as *L. rhamnosus* CRL1505 were shown to induce an earlier and enhanced production of type I IFNs and activation of the inflammatory response in the respiratory tract in response to viral challenges, followed by an improved production of IFN-γ by Th1 cells and an efficient regulation of inflammation (Villena et al., 2012a; Chiba et al., 2013; Zelaya et al., 2014), the preventive nutritional immunobiotic intervention in the “mild/moderate” group could help to avoid the infection or reduce its severity and duration (Figure 5). In addition, immunobiotics could help to diminish community SARS-CoV-2 virus circulation by reducing viral loads in asymptomatic patients. This beneficial effect of immunobiotics could be particularly relevant since it was demonstrated that children tend not to develop severe disease despite being capable of experiencing high viral titers (Kam et al., 2020). Furthermore, immunobiotics could contribute to the reduction of the circulation of other respiratory pathogens such as IFV and *S. pneumoniae* (Figure 4), which increase the risk of mortality in the “severe” group of COVID-19 patients (Chen N. et al., 2020; Ding et al., 2020; Huang et al., 2020; Wang J. et al., 2020; Wu et al., 2020).

On the other hand, the potential beneficial effects of immunobiotics in the “severe” group must be extrapolated more carefully, since in this kind of hosts the immune responses are not only conditioned by the viral infection but also by the alterations associated with the age and/or the presence of other pathologies. As mentioned before, the most important characteristic of the response to SARS-CoV-2 infection in the “severe” group is the impaired innate antiviral response and the subsequent hyperinflammation (Figure 5). The “cytokine storm” and the exuberant inflammation lead to ARDS, disseminated intravascular coagulation, multiple organ failure, and death (Jamilloux et al., 2020). The defective or delayed first line of respiratory antiviral defense, followed by the persistent production of inflammatory cytokines such as IL-6, IL-1β, and TNF-α, results in an increased viral replication and dissemination, desquamation of alveolar cells, hyaline membrane formation, pulmonary edema, impaired clearance of apoptotic cells by dysfunctional macrophages, hemophagocytosis, coagulopathy, and ARDS (Chen N. et al., 2020; Jamilloux et al., 2020; Merad and Martin, 2020; Tay et al., 2020). Immunosuppressive therapies have been proposed for the limitation of the inflammatory damage in severe COVID-19 patients. Individual cytokine antagonists such as blocking antibodies directed to IL-1β or IL-6 were found to be helpful in the recovery of some patients (Jamilloux et al., 2020). However, because of our limited understanding of the inflammatory pathways involved the severe complications of SARS-CoV-2 infection and the wide diversity of patients with different pathologies in the “severe” group, some of these strategies targeting only one cytokine could have detrimental effects in certain type of patients or if they are applied in an incorrect stage of the COVID-19 disease. In contrast, the preventive immunobiotic intervention would induce an earlier influence on several cytokines simultaneously as it has been demonstrated in animal models (Villena et al., 2012a; Chiba et al., 2013; Zelaya et al., 2014). The rapid regulation of inflammatory cytokines and the improvement of IL-10 during the course of respiratory viral infections would allow a reduction of lung tissue damage and could help to delay the appearance of the most harmful effects of the infection, giving more time to clinicians for applying therapies to protect patients from death (Figure 5). In addition, immunobiotics could help to diminish the incidence and/or severity of thrombotic alterations associated to the respiratory viral infection through the modulation of inflammation (Figure 4). In support of this hypothesis, some human clinical trials have demonstrated the ability of immunobiotics to improve the immune health of patients with diseases such as type 2 diabetes, obesity (reviewed in Sáez-Lara et al., 2016), hypertension, and cardiovascular diseases (reviewed in Vasquez et al., 2019). However, much more rigorous clinical and mechanistic studies are necessary to propose immunobiotics as palliatives for the severity of COVID-19 in high-risk patients.

Another potential beneficial effect of the application of immunobiotics in the prevention of SARS-CoV-2 infection is related to their impact on intestinal immunity. Diarrhea is a frequent symptom in 10% of patients infected with SARS-CoV-2 and increasing evidence also indicates possible fecal-oral transmission. Moreover, some patients have diarrhea in the absence of respiratory symptoms, and this may lead to underestimation of COVID-19 cases (reviewed in D'Amico et al., 2020). In this context, considering the ability of some immunobiotic strains to improve the type I IFNs response of intestinal epithelial cells, the activation of intestinal antigen presenting cells as well as to beneficially regulate the inflammatory response (Figure 3; Villena et al., 2012a, 2014; Tada et al., 2016; Albarracin et al., 2017, 2020), preventive nutritional interventions with immunobiotics could help in reducing the incidence and severity of gastrointestinal symptoms and the fecal-oral transmission (Figure 4). In support of this hypothesis, several research works have demonstrated the efficacy of immunobiotics for improving immune responses against coronaviruses in different animals. It was demonstrated that *L. plantarum* Probio-38 and *L. salivarius* Probio-37 were capable of reducing the replication of transmissible gastroenteritis coronavirus (TGEV), a highly contagious enteric pathogen in pigs, in the porcine ST cell line (Rejish Kumar et al., 2010). Similarly, *Enterococcus faecium* NCIMB 10415 reduced the replication of TGEV in ST cells and the improved production of NO, IL-6, and IL-8 were associated to its beneficial effect (Chai et al., 2013). A mixture of probiotic strains was also capable of strengthen the immune system of pigs infected with porcine epidemic diarrhea (PED) coronavirus and improved their reproductive performance (Tsukahara et al., 2018). Interestingly, it was recently reported that a multi-strain probiotic preparation significantly reduced the fecal shedding of the feline coronavirus (FCoV) in cats naturally infected with the pathogen (Addie et al., 2020).

The gastrointestinal alterations in COVID-19 patients can be associated not only to viral replication but also to the disruption of the intestinal microbiota by the antibiotic treatments often necessary in this situation (D'Amico et al., 2020). In this circumstance, it is also plausible that immunobiotics could also be applied for the treatment of COVID-19 patients in order to restore the intestinal microbiota and the optimal function of the immune system. In this regard, the China's National Health Commission recommended the use of probiotics for the treatment of patients with severe COVID-19 in order to preserve intestinal balance and to prevent secondary bacterial infections (Gao et al., 2020).

Finally, an additional important aspect that has to be carefully considered for the potential application of a nutritional immunobiotic intervention in the COVID-19 pandemic is the selection of the most appropriate immunobiotic(s) strain(s). Various probiotic lactic acid bacteria with immunomodulatory activities have been tested in their abilities to improve the resistance to intestinal infections and only a limited number of strains were proved to be effective in the modulation of the antiviral immunity (reviewed in Villena et al., 2016, 2019). Moreover, most of the research related to the beneficial effect of immunobiotics on the host's antiviral immune responses has focused on the intestinal tract while there is less scientific evidence about the capacity of immunobiotics to modulate responses in distant mucosal sites such as the respiratory tract. This is probably related to the fact that immunobiotics with the ability to improve antiviral defenses in both the intestine and the respiratory tract belong to a group with a limited number of members (Villena et al., 2016, 2019; Zelaya et al., 2016). Our own studies have clearly shown that this specific immunomodulatory property is a strain-dependent characteristic. In our hands, immunobiotic strains such as *L. plantarum* CRL1506 that is capable to stimulate the antiviral intestinal immunity (Villena et al., 2012a, 2014; Tada et al., 2016; Albarracin et al., 2017) was not able to influence respiratory antiviral immune responses after its oral administration (Villena et al., 2012a; Chiba et al., 2013; Zelaya et al., 2014). Therefore, the immunobiotic strains proposed to strengthen the population's immune system during viral epidemics or pandemics should have a strong scientific background that clearly supports their ability to modulate the mucosal antiviral immunity. In addition, the selection of new antiviral immunobiotic strains that have different biotechnological properties could enhance the development of various types of functional foods that could be used in nutritional immunobiotic interventions around the world to reduce the incidence and severity of intestinal and pulmonary infections caused by viruses such as the SARS-CoV-2. In this regard, our recent *in vitro* and *in vivo* studies demonstrated that *L. plantarum* MPL16 modulated the intestinal and respiratory antiviral immunity in a similar way as the CRL1505 strain (Albarracin et al., 2020). Whereas, *L. rhamnosus* CRL1505 has been used mainly in dairy functional products, the MPL16 strain has shown a remarkable ability to growth and ferment wakame (*Undaria pinnatifida*) that is the most popular edible brown algae in Asian countries. Then, the different biotechnological properties of immunobiotics such as *L. plantarum* MPL16 could potentiate the development of non-dairy functional foods with the ability to improve antiviral immunity in the intestine and the respiratory tract.

## Conclusions

As the SARS-CoV-2 pandemic expands, scientists have made great advances in the knowledge of the genome characteristics and the infection biology of this virus that will allow the development of new vaccines and therapeutics in the future. However, in order to favorably affect the trajectory of this pandemic and mitigate as much as possible its detrimental effect on public health and economy, all the available scientific resources should be put at the service of the community. More than a decade of research investigating the cellular and molecular mechanisms involved in the improvement of respiratory antiviral defenses by beneficial immunobiotic microorganisms clearly indicate their potential to favorably influence the immune response against SARS-CoV-2 virus. The demonstration of ability of beneficial microorganisms to enhance type I interferons and antiviral factors in the respiratory tract, stimulate Th1 response and antibodies production, and regulate inflammation and coagulation activation during the course of viral infections reducing tissue damage and preserving lung functionally, provide solid scientific basis for proposing immunobiotic nutritional interventions to help in the strengthening of the immune system and the reduction of the incidence and severity of COVID-19. In addition, taking into consideration that scientists speculate that this new virus will accompany humanity from now on, studies in experimental animals as well as clinical trials that conclusively demonstrate the beneficial effect of immunobiotics on SARS-CoV-2 infection, particularly in high-risk populations, could enhance their application to mitigate the virus-associated detrimental effects of the immune system. If this approach is scientifically validated, rapid repurposing of immunobiotics such as *L. rhamnosus* CRL1505 or *L. plantarum* MPL16 will be effective and timely in the fight against COVID-19.

## Author Contributions

JV and HK wrote and revised the manuscript. All authors contributed to the article and approved the submitted version.

## Conflict of Interest

The authors declare that the research was conducted in the absence of any commercial or financial relationships that could be construed as a potential conflict of interest.
